# Supplementary material for: Modeling glioblastoma heterogeneity as a dynamic network of cell states
Source: Mol Syst Biol. 2021 Sep 16;17(9):e10105. doi: 10.15252/msb.202010105 (PMC8444284; doi:10.15252/msb.202010105)
Supplement: Supplementary file 5 — Source Data for Figure 3 [file MSB-17-e10105-s001.zip › Figure3A_sourcedata/GSEA_3065/hallmarks_state5.GseaPreranked.1623416302988/HALLMARK_PROTEIN_SECRETION.html]

Details for gene set HALLMARK\_PROTEIN\_SECRETION[GSEA]

|  || Dataset | state5 |
| Phenotype | NoPhenotypeAvailable |
| Upregulated in class | na\_pos |
| GeneSet | HALLMARK\_PROTEIN\_SECRETION |
| Enrichment Score (ES) | 0.28509915 |
| Normalized Enrichment Score (NES) | 1.1231154 |
| Nominal p-value | 0.22134387 |
| FDR q-value | 0.27833322 |
| FWER p-Value | 0.923 |
Table: GSEA Results Summary

  

Fig 1: Enrichment plot: HALLMARK\_PROTEIN\_SECRETION      
 Profile of the Running ES Score & Positions of GeneSet Members on the Rank Ordered List

  

| PROBE | GENE SYMBOL | GENE\_TITLE | RANK IN GENE LIST | RANK METRIC SCORE | RUNNING ES | CORE ENRICHMENT || 1 | CD63 |  |  | 24 | 0.769 | 0.0401 | Yes |
| 2 | LAMP2 |  |  | 69 | 0.584 | 0.0676 | Yes |
| 3 | LMAN1 |  |  | 95 | 0.533 | 0.0944 | Yes |
| 4 | TMED10 |  |  | 131 | 0.498 | 0.1182 | Yes |
| 5 | TMX1 |  |  | 255 | 0.406 | 0.1269 | Yes |
| 6 | DST |  |  | 313 | 0.376 | 0.1414 | Yes |
| 7 | ATP1A1 |  |  | 334 | 0.366 | 0.1595 | Yes |
| 8 | YIPF6 |  |  | 340 | 0.364 | 0.1791 | Yes |
| 9 | CTSC |  |  | 347 | 0.362 | 0.1986 | Yes |
| 10 | CLCN3 |  |  | 356 | 0.360 | 0.2177 | Yes |
| 11 | CLN5 |  |  | 497 | 0.312 | 0.2193 | Yes |
| 12 | ADAM10 |  |  | 574 | 0.292 | 0.2270 | Yes |
| 13 | RER1 |  |  | 606 | 0.283 | 0.2392 | Yes |
| 14 | M6PR |  |  | 641 | 0.277 | 0.2508 | Yes |
| 15 | GLA |  |  | 654 | 0.274 | 0.2647 | Yes |
| 16 | IGF2R |  |  | 705 | 0.260 | 0.2736 | Yes |
| 17 | PPT1 |  |  | 744 | 0.251 | 0.2833 | Yes |
| 18 | PAM |  |  | 844 | 0.233 | 0.2851 | Yes |
| 19 | ERGIC3 |  |  | 1457 | 0.157 | 0.2250 | No |
| 20 | COPB1 |  |  | 1492 | 0.153 | 0.2297 | No |
| 21 | VPS45 |  |  | 1530 | 0.149 | 0.2338 | No |
| 22 | MON2 |  |  | 1541 | 0.148 | 0.2409 | No |
| 23 | EGFR |  |  | 1547 | 0.147 | 0.2485 | No |
| 24 | STX16 |  |  | 1718 | 0.132 | 0.2367 | No |
| 25 | SCAMP3 |  |  | 1807 | 0.126 | 0.2339 | No |
| 26 | SCAMP1 |  |  | 1816 | 0.126 | 0.2399 | No |
| 27 | ATP6V1H |  |  | 2034 | 0.109 | 0.2216 | No |
| 28 | ABCA1 |  |  | 2142 | 0.103 | 0.2153 | No |
| 29 | TMED2 |  |  | 2321 | 0.091 | 0.2003 | No |
| 30 | DNM1L |  |  | 2401 | 0.085 | 0.1962 | No |
| 31 | ATP7A |  |  | 2505 | 0.079 | 0.1890 | No |
| 32 | SEC24D |  |  | 2586 | 0.074 | 0.1841 | No |
| 33 | USO1 |  |  | 2636 | 0.072 | 0.1826 | No |
| 34 | ARFGEF2 |  |  | 2739 | 0.066 | 0.1748 | No |
| 35 | SEC31A |  |  | 2963 | 0.054 | 0.1527 | No |
| 36 | STX12 |  |  | 3167 | 0.043 | 0.1323 | No |
| 37 | CLTC |  |  | 3513 | 0.027 | 0.0950 | No |
| 38 | COG2 |  |  | 3569 | 0.024 | 0.0902 | No |
| 39 | SNAP23 |  |  | 3621 | 0.023 | 0.0857 | No |
| 40 | KIF1B |  |  | 3759 | 0.017 | 0.0712 | No |
| 41 | GBF1 |  |  | 3822 | 0.015 | 0.0651 | No |
| 42 | SEC22B |  |  | 3938 | 0.010 | 0.0527 | No |
| 43 | STX7 |  |  | 3940 | 0.010 | 0.0531 | No |
| 44 | RPS6KA3 |  |  | 4037 | 0.006 | 0.0426 | No |
| 45 | ARFGEF1 |  |  | 4041 | 0.006 | 0.0426 | No |
| 46 | VAMP4 |  |  | 4153 | 0.001 | 0.0302 | No |
| 47 | AP1G1 |  |  | 4300 | -0.006 | 0.0141 | No |
| 48 | GOSR2 |  |  | 4433 | -0.011 | -0.0001 | No |
| 49 | TOM1L1 |  |  | 4506 | -0.014 | -0.0074 | No |
| 50 | SSPN |  |  | 4582 | -0.017 | -0.0149 | No |
| 51 | AP2B1 |  |  | 4612 | -0.019 | -0.0171 | No |
| 52 | STAM |  |  | 5090 | -0.037 | -0.0687 | No |
| 53 | VPS4B |  |  | 5105 | -0.037 | -0.0682 | No |
| 54 | ARFIP1 |  |  | 5411 | -0.049 | -0.0998 | No |
| 55 | AP3B1 |  |  | 5431 | -0.049 | -0.0992 | No |
| 56 | OCRL |  |  | 5434 | -0.049 | -0.0967 | No |
| 57 | MAPK1 |  |  | 5469 | -0.051 | -0.0976 | No |
| 58 | NAPG |  |  | 5561 | -0.056 | -0.1048 | No |
| 59 | COPB2 |  |  | 5597 | -0.057 | -0.1055 | No |
| 60 | GOLGA4 |  |  | 5615 | -0.058 | -0.1042 | No |
| 61 | ICA1 |  |  | 5718 | -0.063 | -0.1122 | No |
| 62 | ZW10 |  |  | 6365 | -0.093 | -0.1796 | No |
| 63 | BET1 |  |  | 6654 | -0.111 | -0.2058 | No |
| 64 | ANP32E |  |  | 6655 | -0.111 | -0.1996 | No |
| 65 | RAB22A |  |  | 6715 | -0.115 | -0.1999 | No |
| 66 | VAMP7 |  |  | 6717 | -0.115 | -0.1936 | No |
| 67 | CAV2 |  |  | 6984 | -0.135 | -0.2160 | No |
| 68 | NAPA |  |  | 7167 | -0.152 | -0.2280 | No |
| 69 | RAB5A |  |  | 7179 | -0.154 | -0.2207 | No |
| 70 | TPD52 |  |  | 7309 | -0.168 | -0.2259 | No |
| 71 | SNX2 |  |  | 7424 | -0.184 | -0.2284 | No |
| 72 | SCRN1 |  |  | 7434 | -0.185 | -0.2192 | No |
| 73 | ARFGAP3 |  |  | 7555 | -0.198 | -0.2217 | No |
| 74 | AP3S1 |  |  | 7728 | -0.223 | -0.2286 | No |
| 75 | RAB9A |  |  | 7740 | -0.224 | -0.2174 | No |
| 76 | YKT6 |  |  | 7874 | -0.249 | -0.2185 | No |
| 77 | RAB14 |  |  | 7889 | -0.252 | -0.2061 | No |
| 78 | TSG101 |  |  | 7995 | -0.275 | -0.2026 | No |
| 79 | ARCN1 |  |  | 8043 | -0.284 | -0.1921 | No |
| 80 | VAMP3 |  |  | 8102 | -0.298 | -0.1821 | No |
| 81 | BNIP3 |  |  | 8255 | -0.341 | -0.1803 | No |
| 82 | RAB2A |  |  | 8273 | -0.346 | -0.1630 | No |
| 83 | ARF1 |  |  | 8606 | -0.495 | -0.1727 | No |
| 84 | COPE |  |  | 8721 | -0.583 | -0.1532 | No |
| 85 | AP2M1 |  |  | 8743 | -0.600 | -0.1222 | No |
| 86 | CLTA |  |  | 8794 | -0.646 | -0.0919 | No |
| 87 | AP2S1 |  |  | 8799 | -0.648 | -0.0563 | No |
| 88 | GNAS |  |  | 8822 | -0.674 | -0.0213 | No |
| 89 | SOD1 |  |  | 8848 | -0.709 | 0.0153 | No |
Table: GSEA details [plain text format]

  

Fig 2: HALLMARK\_PROTEIN\_SECRETION: Random ES distribution      
 Gene set null distribution of ES for **HALLMARK\_PROTEIN\_SECRETION**
